# Identification of a novel monogenic autoinflammatory disease due to mutation in a mitochondrial chaperone protein in a single kindred, and cure with allogeneic haematopoietic stem cell transplantation

**DOI:** 10.1186/1546-0096-12-S1-O22

**Published:** 2014-09-17

**Authors:** Ariane Standing, Coro Paisan-Ruiz, Despina Eleftheriou, Ying Hong, Ebun Omoyinmi, Dorota Rowcenzio, Helen Lachmann, Philip Hawkins, Patricia Woo, Nigel Klein, Paul Brogan

**Affiliations:** 1 UCL Institute of Child Health, London, UK; 2 UCL Institute of Neurology, London, UK; 3 National Amyloidosis Centre, UCL Medical School, London, UK; 4 UCL, London, UK

## Introduction

The monogenic autoinflammatory syndromes are characterised by seemingly unprovoked inflammation which derives from a disruption of innate immunity. Novel as yet undefined autosomal recessive syndromes are increasingly recognised in consanguineous families. This type of family is ideal for genetic mapping.

## Objectives

To conduct genetic mapping and sequencing to identify the causal variant(s) in a single consanguineous family with a severe unclassified autoinflammatory disease; and confirm pathogenicity with functional studies of novel genetic mutation(s).

## Methods

Three affected children in a Pakistani family suffered from a severe and unusual autoinflammatory syndrome, presenting in the first year of life with recurrent fevers, erythema nodosum-like rash, severe oromucocutaneous ulceration, systemic inflammation, and massively elevated serum IgD, without mutation in MVK. One of the affected children also suffered from multifocal sterile osteomyelitis with bony lytic lesions and died at age 12 months from bronchopneumonia, and acute cervical myelopathy from cervical vertebral collapse. The two older children were resistant to treatment with corticosteroids, colchicine, several different DMARDs, anakinra and infliximab. Both were cured by allogeneic haematopoietic stem cell transplantation (HSCT) in their teenage years and remain well and off all treatment approximately 5 years later. DNA from the three patients, two unaffected siblings and their parents were genotyped on Illumina 610 SNP arrays and this data was used for homozygosity mapping and parametric multipoint linkage analysis. A 5Mb region was identified from this mapping. Candidate genes were chosen by members of an expert panel and the exons of these genes were Sanger sequenced. DNA from the entire homozygous region linked to the disease locus (5Mb) was captured using a custom designed 385k capture array from Nimblegen and then resequenced using the Illumina Genome Analyser II, revealing over 50 coding change variants. These entered a filtering process involving: the selection of rare variants and screening of extended family members, ethnically matched controls from the Jat Kalyal tribe, and the exclusion of unlikely candidates. siRNA knockdown was conducted in THP1 monocytic cells derived to macrophages with measurement of cytokine and reactive oxygen species (ROS) production measured by flow cytometry and electron spin resonance (ESR).

## Results

A missense variant of interest in a mitochondrial chaperone-like protein was discovered that fully segregated with disease in the family. Knockdown of this protein in THP1-derived macrophages caused an enhanced production of mitochondrial superoxide detectable by ESR and mitoSOX fluorescence, and increased production of TNF-alpha.

## Conclusion

We describe a novel monogenic autoinflammatory disease driven by mitochondrial ROS production, leading to enhanced inflammatory responses. Increased leucocyte ROS have previously been implicated in other autoinflammatory diseases (TRAPS, FMF, CAPS and FCAS2); we suggest that this was the cause of the severe, recalcitrant and novel autoinflammatory syndrome in the family described herein, ultimately cured by HSCT.

## Disclosure of interest

None declared.

